# Suggestion to Manager Abbey

**Published:** 1881-04

**Authors:** 


### A SUGGESTION TO MANAGER ABBEY.

If Mr. Abbey will but abandon this "Passion Play" and in its stead produce "The Evolutive Creation," not according to Moses, but Darwin and the other great evolutionists and materialists, he will both amuse and instruct. Something thus:

Scene 1—Elementary chaos, wild music, thunder, lightning, meteors, comets, aerolites and earthquakes rushing to and fro over the stage; gongs, guns and drums; gas turned off at every alternate second and house left in total darkness; sky rockets, bombs, blue fire, red fire, green fire, yellow fire and firecrackers; terrific explosion; stage blown up through the roof; elementary chaos.

Scene 2—Grand and profound elementary silence for fifteen minutes. The elements are now combining. Crystallization and development of organization. N. B.—The male portion of the house may take this opportunity to go out and see a coming man.

Scene 3—Birth of the first protoplasm. Powerful calcium light turned to a focus on the stage. Audience provided with microscopes. It wriggles! Protoplasm! The first life is born! Thrown by a ten thousand magnifying reflector on a white curtain.

Scene 4—Evolution by successive stages of protoplasm to a clam. Little Neck. Several planets arrive. The clam organizes the planetary system. Arrests a booming comet in its mad career and creates the sun. Evolution of the stars. Represented by several stage stars on the stage.

(Wagnerian music.)

Scene 5—The clam evolutes to an oyster. Saddle Rock. The oyster regulates the sun's rising and setting. Sun organizes a staff of planets on regular salaries.

(Triumphant music.)

Scene 6—Evolution. Aspirations of the oyster. It desires to climb a tree. Song, "Rise up, William Riley." Insertion of a new clause in the Constitution of the universe. The oyster puts forth claws and becomes a turtle. Combat between the turtle and the oyster. Great battle of Armageddon. Principle of good prevails. Turtle wins.

Scene 7—Pause of 9,000,000,000 years. Turtle show evolutes. Suddenly the grand imprisoned forces of nature within its shell assert themselves. The turtle rises on its hind legs, takes off its coat and stalks out of its shell the primitive man! Creation is accomplished. Air, "Johnny Comes Marching Home." Elections, newspaper, rail and steamboat accidents succeed in the grand order of the Universe. Curtain.

---

Drinking Blood.—It is said that between 200 and 300 men and women of St. Louis drink daily from a half to a pint of blood piping hot from the veins of slaughtered cattle. More blood drinking by consumptives and aged persons is done in September and October than during the remainder of the year. The blood of young steers is the best, and should be caught as it comes from the animal, and should be drunk while the foam is still on and the steam rising. Consumptives are advised, in addition to drinking the blood, to sit in a slaughter-house for a couple of hours each day at killing time to inhale the "steam" of the running blood.

### EBONIZED WOOD.

A very simple process for ebonizing wood is given in the *Art Interchange*, as follows:

"The wood is first stained with a decoction of logwood, which may be purchased from any druggist. It is dissolved in warm water until all has been taken up that the water will hold. Application to the wood is made freely, with a large soft bristle brush, and the surface is rubbed with a cloth to prevent the formation of a gummy coat thereon. After the article has been left to dry for a few hours, the second application, which consists of vinegar in which a quantity of nails or clean filings have been soaked for several days, is also freely laid on with a brush. The moment the vinegar touches the wood it combines with the logwood solution in the pores, making an ink which is a permanent jet black stain. The influence of the iron in the vinegar is all important. If any tendency to grayness is noticed, a second treatment is necessary; but this seldom happens. When perfectly dry the article is varnished and rubbed down or finished with furniture oil well rubbed in. Cherry is considered the best wood for ebonizing. Whitewood, maple and beech are used with good effect. Any close grained, dense wood will answer—ash, chestnut and oak are not suitable. This process, I am told, is used for fine ebony and gold furniture."

### POINTEDLY PUT.

Says the Boston *Transcript*: "You put blinders on a horse so he can see nothing that is going on about him, and then blame him for trembling and starting to run for every little noise. You forget how frightened you were when, with blinded eyes, you were initiated into the awful mysteries of the High Mightiful Lodge of Unbiased and Superincumbent Chinwaggers. And why shouldn't a horse be frightened as easily as a donkey.

"Some Quacks" is the title of an amusing article in *Scribner's Magazine* for February. An office-boy to a "root-doctor" relates that he was once sent into the woods to get some of the inner bark of the butternut tree. "Tom," said the doctor, as he departed, "I want you to scrape this bark downward. It is for a cathartic. Don't scrape it upward, or it will be an emetic. And whatever you do, Thomas, do't scrape it both ways. If you do, nobody on earth can tell how it will act."

